# Exploring enablers and barriers to breast self-examination among women in the North Shewa Zone, Oromia, Ethiopia: a qualitative study

**DOI:** 10.1038/s41598-023-44808-x

**Published:** 2023-10-14

**Authors:** Dursa Hussein, Ketema Gashaw, Tinsae Abeya Geleta, Derara Girma, Leta Adugna Geleta, Befekadu Tesfaye Oyato

**Affiliations:** 1 Department of Public Health, College of Health Science, Salale University, Fitche, Ethiopia; 2 Department of Midwifery, College of Health Science, Salale University, Fitche, Ethiopia

**Keywords:** Health care, Medical research

## Abstract

Breast cancer (BC) is the leading cause of cancer death worldwide and the second most common cancer overall. Breast self-examination (BSE) is one of the cheapest methods used for the early detection of BC in asymptomatic women. More than 90% of cases of BC can be detected by women themselves. This reality stresses the importance of BSE as the key BC detection mechanism. However, in Ethiopia, most of the BE studies were not conducted among women in the general population. Therefore, this study aimed to explore enablers and barriers to breast self-examination among women in the North Shewa Zone, Oromia. A descriptive qualitative study design was conducted from August 1, 2022, to September 30, 2022. Five focus group discussions (FGDs) were conducted with 46 women from one selected district in the North Shewa Zone, Oromia. A Purposive sampling technique was used to select participants for FGD. The audio-recorded data were transcribed verbatim to “Afan Oromo”. Transcribed data were translated into English. The data were manually coded into themes and analyzed manually by using inductive thematic analysis. The findings of the study were discussed under five themes of enablers and three themes of barriers. The five themes of enablers were knowledge about BC, knowledge about BSE, experience of BSE practice, perceived susceptibility, and perceived benefit of BSE practice. The four themes of barriers were low knowledge of BSE practice, misconceptions about BSE practice, and fear of detecting BC. These findings suggest that targeted health education programs, collaboration between healthcare providers and local stakeholders, and the availability of support services can play a crucial role in overcoming barriers and encouraging BSE practice for early detection of breast abnormalities.

## Introduction

Breast self-examination (BSE) is one of the screening methods, which involves the woman looking at herself and feeling each breast for possible lumps, distortions, or swelling^1–3^. More than 90% of cases of breast cancer (BC) can be detected by women themselves^4^. This reality stresses the importance of BSE as the key BC detection mechanism^4^. Nowadays, BC is a major women’s health problem globally. Meanwhile, primary prevention for BC is still available^5,6^ New cases of BC worldwide are estimated at 252,710 with almost 459,000 related deaths^7^. According to the American cancer society (ACS), 1 out of 8 United States women experiences BC in their lifetime. ACS has predicted the incidence of BC in women around the world to reach around 3.2 mil new cases per year in 2050^1^.

According to the reports of Global Burden of Cancer (GLOBOCAN) in 2020, of the 19.3 million new cases of cancer globally, BC accounted for 24.5% of which 16.8% occurred in Sub-Saharan Africa (SSA) and also accounted for 15% of the 9.9 million mortality due to cancer worldwide, while in SSA accounting for 12.1%^8,9^. In Ethiopia, in 2020 the number of new cases of cancer among females of all age were 50,598 while, BC accounts for 31.9% and also, cancer accounts for about 5.8% of total national mortality while, BC accounts for 17.5%^10^. In Ethiopia, BC incidence is rising and becoming the foremost common cancer, causing high rates of morbidity and mortality^10^. The incidence of BC accounts for 15,244 (22.6%) of all cases of cancer and 8,159 (17.5%) cancer mortality annually^11^.

WHO recommends cancer prevention as an essential component of all cancer control plans because about 40% of all cancer deaths can be prevented^12^. BSE has been promoted for many years as a screening method for BC at an early stage, to decrease the risk of dying from BC^12^.

BSE practice has been valued to enable women to take concern their health and it is a suitable and cheap means that can be implemented regularly^13^. A country with inadequate resource facilities and poor health systems has to promote early diagnosis programs based on BSE, awareness of early signs and symptoms, and prompt referral to diagnosis and treatment^14^.

The practice of BSE has been reported in different countries. 12.5% in Indonesia^15^, 11% in Yemen^16^, 4.0% in Saudi Arabia^17^, 37.6% in Ghana^18^, 15% in Cameroonian^19^, 24.4% Nigeria^20^ 13.2% in Bale zone^21^, 15% in Jimma^22^, 51.4% in Adama^4^, 45.8% in Gondar^23^, and 6.25% in Adawa^24^ town women had performed BSE on regular basis (monthly).

Factors affecting the practice of BSE were reported from different countries. These are age, family history of BC, knowledge of BSE, level of education, and women's perception towards BSE practice and BC^15,17,18,24–28^. BSE is the only feasible approach, as it is a cheap and easily applicable method across a wide population. Its ultimate purpose is early detection and treatment. However, poor practice among women has been a major obstacle to its effectiveness. Therefore, increasing women's practice of BSE through the creation of BC awareness campaigns is crucial^4,15–17,21,22^.

In Ethiopia, most of the BSE studies were not conducted among women in the general population and have been conducted on university students^29,30^, health care providers^31–34^, and urban populations^4,23^ and also not added qualitative approaches which is more important to understand health-seeking behaviour or why people not utilizing health service^4,21,35^. Due to this, many women miss early detection, as well as treatment opportunities Thus, this study aimed to exploring enablers and barriers to breast self-examination among women in the Kuyu district.

## Methods

### Study design, period, and setting

A descriptive qualitative study design was conducted from August 1, 2022 to September 30, 2022. In the Kuyu district. The district is found in the North shewa zone, and composed of 26 rural kebeles and 4 urban kebeles. According to the Ethiopian context, a kebele is the smallest administrative unit below the district. The district is located in the Northern part of Ethiopia, 38 km from the capital city of zone Fiche and 156 km from the capital city of the country Addis Ababa. According to the Kuyu District Health Office estimate in 2021/22, the total population in the district is 180,180, with 8321 of females, and the estimated number of reproductive age women is 39,874. The district has 1 General hospital, 7 health centers, and 27 functional health posts regarding public health facility coverage^36^.

### Population

Purposely selected reproductive-age women (15–49) living in the selected kebeles of Kuyu district. The authors judged to include diversified participants in order to elicit rich ideas and insight into the issue considering some factors such as their educational level, residence, marital status and age.

### Sample size and sampling procedures

A total of 46 women were involved in five FGDs. FGDs were conducted in one districts of the North Shewa zone namely: Kuyu, which included 6 to 10 members in each group. From each selected kebele, one groups of reproductive-age women and one group of students from the selected district, included in the FGD.

### Data collection tools, personnel, and procedures

Data were collected in the local language (Afan Oromo) by using semi-structured interview guides. The interview guide was developed after reviewing relevant literatures and considering the objective of the study^21,29,37,38^. The guides were prepared in English by the principal investigator (PI), translated into Afan Oromo, and checked by experts for more clarity. The FGDs guides had a list of a few discussion points such as knowledge about BC and BSE, barriers to BSE practice and enablers of BSE with several follow-up probes used to capture beneath of the issue. The FGDs were moderated by an experienced health professional and note-taker. During the discussion, the audio-recorded data were transcribed verbatim to “Afan Oromo” by the PI. Finalized transcribed data were correlated with field notes prior to translation. Transcribed data were translated to English. The data were manually coded and themes. Inductive approach was used while formulating codes and themes from the data obtained. The investigators drew codes and themes jointly through discussion.

### Data quality assurance

To ensure the quality of data, the PI considered trustworthiness it is the fundamental criterion for qualitative study and finding considered rich in information for the purpose of saturating data.

### Credibility (related to internal validity)

Credibility depends upon how closely the collection, presentation and interpretation of data match the underpinning philosophy of the research methodology chosen to address the research question^39^. So, to maintain the credibility, of the research findings FGD guidelines were evaluated by the professionals, before the data collection. For the two individuals who participated in the FGD, orientation about the purpose of the FGD and responsibility was given before the FGD takes place to avoid unnecessary interruption and keep the rights of the participants. Triangulations were made by using multiple data sources and diversifying the study participants in terms of age, residence, and educational level for a deeper understanding of the BSE practice.

### Transferability (related to generalizability)

Transferability is about providing enough information in accessible language to enable another to answer the question to transfer in another setting^39^. To maintain the transferability of the finding, appropriate probes were used to obtain detailed information on responses. Detailed field notes and digital audio recordings were done for all FGD before and during analysis (thick description).

### Dependability (related to reliability)

Dependability can be described as making sure research questions were clear and appropriate to the study design, ensuring transparency of the researcher`s role and the use of appropriate data collection^39^. So, to maintain the dependability of the finding the research process member checking was made by returning the preliminary findings to the participants to correct errors and challenge what was perceived as wrong interpretations. The prolonged meeting was made to address individuals with different ideas acceptance was built with participants. Furthermore, the interpretations of the researcher were challenged through discussion of the preliminary analysis in group meetings with groups of data collectors and the researcher`s supervisors.

### Conformability (related to objectivity)

To ensure Conformability of the finding the FGD guidelines were followed to make them homogeny in terms of age, educational level and residence and the discussion process were conducted by bilingual individuals (two female) to make the participant freely react to the issue under discussion. Detailed field notes and digital audio recordings were done for all FGD and data analysis in each sub-study was crossed checked and the results were reviewed about themes and subthemes with which their original data were linked by the investigator and at least one other researcher.

### Data process and analysis

Data, during FGD were first transcribed in the Afan Oromo language, in which the discussion were conducted. Next, the data were translated and transcribed into English by senior language experts at our university. Then, a final edition of the code was developed, and the categories and themes were constructed. Data were coded, categorized, and analysed manually by using inductive thematic analysis (by organizing the topics raised at the time of the in-FGD), and the result was presented in narrative forms to describe barriers and enablers of breast self-examination.

### Ethical approval

The study protocol was approved and ethical approval letter is provided by the Ethical Review Committee of Salale University with reference number IRB/878/14. The study was performed in line with the World Medical Association Declaration of Helsinki on medical research. Written informed consent was obtained from every study subject before the discussion was started and Minor assent or parental permission for the study subject less than 18 years old. The participant’s right to refuse to participate in the interview, not respond to any questions, and to leave at any time was also preserved.

## Results

### Description of participants

A total of 46 women were involved in five FGDs. Participants’ ages ranged from fifteen to forty-nine. Most of the participants were between the ages of 25–34 years. A majority of them were secondary education in terms of educational status and housewife in occupational status Each participant showed good interest in the topic and gave enthusiastic answers to the questions (Table 1).

**Table 1:** Sociodemographic characteristics of the study participants to explore enablers and barriers to BSE practice among reproductive age women in the North Shewa Zone Oromia 2022.

Variables	Categories	Frequency	Percent (%)
Age	15–24	8	17.4
	25–34	24	52.2
	35 and above	14	30.4
Residence	Urban	24	52.2
	Rural	22	47.8
Marital status	Single	12	26.1
	Married	14	30.4
	Widowed	12	26.1
	Divorced	8	17.4
Educational level	Primary education	8	17.4
	Secondary education	20	43.5
	College and above	18	39.1
Occupation	Housewife	17	37.0
	Student	10	21.7
	Government employed	9	19.6
	Marchant	10	21.7

### Enablers to BSE practice

The findings of this study were discussed under five major themes: Knowledge about BC, knowledge about BSE, experience of BSE, Perceived benefits of treatment practice and Perceived susceptibility.

#### Theme 1: knowledge about BC

As confirmed from all FGD as low awareness about BC. But among them those who performing BSE raised that as they had heard about BC from media (Television and Radio) and BC patients and they know exactly what it was., especially its sign and symptoms, risk factors, screening methods and management. The majority of the women agreed that screening methods for BC.

Some of their responses were:

BSE is useful to understand the normal look and feel of your breast so, by doing it you can identify any problems in your breast early. (Women 12, Group 1–5)

BC is a serious and fatal disease. I am scared that I suffer from BC. It is a painful lump and gradually increases in size and pain intensity so to identify on time by BSE is useful for the quality of life (Women 10, Group 1–5).

In our setting, there has been awareness creating activities or education by doctors on breast matter (presence of the disease, its consequences, its symptoms and its option of treatment).” We have heard also some information on television and radio. Some of us are hearing even the presence of its screening methods and methods for early detection of breast cancer today from them. (women16, Group 1–5).

#### Theme 2: knowledge about BSE

As, confirmed from the discussion out of 46 participants in this discussion, 22 women had practiced BSE at least one during the past 6 months. Those women who had performed BSE answered that *“we know how to do it”, “we know screening methods” and “we believe it important for early diagnosis and treatment”.*

I heard about BSE and I know the way to do it (women 14, Group 1–5).

I heard about BSE and accepted as it important for early detection of BC and I know the right time to perform as well as its technique (women 8, Group 1–5).

BSE is squeezing of the breast thoroughly with hand in standing In front of the mirror (women 14, Group 1–5).

I heard from Health professionals as early detection of BC improves the chance of survival, do to that I do it every month” (women 14, Group 1–5).

I wish I had the knowledge to do so. (Women 6, Group 1–4).

#### Theme 3: experience of BSE practice

As confirmed from the discussion the majority of women need to touch their breasts if they doubt there is problems and also the majority of them believe performing BSE is important for early detection of any breast diseases. Most of the women stated that BSE is known among us. *“BSE is useful to understand the normal look and feel of your breast so, by doing it you can identify any problems in your breast early”.* (Women 17, Group 1–5).

#### Theme 4: perceived susceptibility

The majority of participants perceived that the cause of BC is hereditary, supernatural power and a few associated it with breast enlargement in the childhood period. Those women who had performed BSE answered that they have a family history of BC, perceived they are susceptible to the disease and perceived the severity of the disease. In this discussion, the majority of the women expressed that they perceived the risk of BC. However, the participants varied in assessing their risk of BC. The majority of them believed that their risks were connected with having a family history of BC. Their responses are as follows:

My mother was diagnosed with a BC last five years starting from that time. I was nervous about having BC due to that I checked my breast every month. (Women 5, Group 1–4).

I was nervous about having BC because my mother had BC, and had a mastectomy for one of her breasts last year. Also, I have sometimes felt the pain that is why I want to examine my breast every month. (Women 3, Group 1–4).

#### Theme 5: perceived benefits of BSE practice

Regarding believing it is important for early diagnosis and treatment, most participants in this discussion know well that BSE can help find the breast problem because their relatives and friends found the BC by self-examination. BSE evidently works, as the following examples state:

My friend discovered her BC by touching her own body. Unfortunately, her tumour been growing. If it had been found earlier, everything could be different. I think that every woman should do BSE. (Women 13, Group 1–4).

I want to say I did BSE just for health, not intending to argue with the size of my breasts. The incentive of health is still strong (laughs). By the way, I have recommended the skills to my friends. For example, I told my boy-boyfriends I could teach his sister; as we are all women, we have to protect ourselves… (Women 2, Group 4).

The Kuyu General Hospital caregiver found the lump in my mother's breast when she examined my mother. She did my mother a great favour. …This is a good chance for me to learn these skills. I think that I can do it more often with my fingers so that it might keep my mind at ease. (Women 2, Group 1).

### Barriers to BSE practice

Barriers to practice BSE were explored and categorized under four themes as follows:

#### Theme 1: low knowledge of BSE practice

As confirmed from the discussion as there is a lack of BSE practice. The women who had never performed BSE answered that they did not know how to do it correctly, they didn’t know what to find and they didn't understand if the mass they detected was normal or not. Their responses were as follows:

Just we heard about BSE as one method of BC screening. But it is not sure how to do it and doesn’t know the right time to perform it (women 26, Group 1–4).

I heard about BSE. But not know the way to do (women 14, Group 1–2).

I heard about BSE and accepted as it important for early detection of BC but I did not know the right time to perform as well as its technique (women 24, Group 1–3).

BSE is squeezing of the breast thoroughly with hand in sitting position at the time of bathing, but not the axilla because of BC (women 4, Group 1–2).

Because I do not know how to do it, and I have no extra time to do it. (Women 5, Group 1, 3).

#### Theme 2: misconception about BSE practice

Almost, confirmed from the discussion as there is a misconception about BC and BSE practice. The women who had never performed BSE answered that, have no history of a breast lump, and have no symptoms, they did not believe it important and fear made them afraid to talk about the practice of BSE. Their responses were as follows:

I have no history of breast disease. I do not feel pain in my breast. I think that I do not need to do a breast examination. (Women 12, Group 1–3).

I rarely perform it because I have no problem with breast and usually visit health facilities and HCWs to get a child. Nevertheless, I have never asked about BC, and also, they do not suggest me to perform BSE (Women 5, Group 1–4).

, I heard about BSE But it is not sure how to do it so, don’t believe as it important for early detection of breast disease. (Women 15, Group 1–4).

BSE practice, I feel it is not necessary to me (laugh). As you saw I am an adult, I am married and have given birth to children and fed breast to all of my children so, through all this time I do not feel any changes or problems with discharge, and no pain and if this was needed, it should be done by a healthcare professional. (Women 9, Group 1–3).

I did BSE but I couldn´t find anything, I mean I couldn´t figure it out. Then, I asked myself why I am breaking me down and I gave up practicing these self-examinations. (Women 19, Group 1–4).

We don’t want to talk about BC because when you talk about a disease, the spirit of that disease can inflict or make it happen to you. It is a disease that makes them remove your breast? It is scaring (Women 5, Group 1–4).

Frequent breast examination will make one detect a growth. The breast is a private area that should be kept as such. (Women 5, Group 2–4).

#### Theme 3: fear of detecting BC

As confirmed from the discussion as there is a lack of BSE practice. Those women who had never performed BSE answered that they did not touch their breast, and they don’t want to talk about the diseases. Their responses were as follows:

We don’t want to talk about BC because when you talk about a disease, the spirit of that disease can inflict or make it happen to you. It is a disease that makes them remove your breast? It is scaring (Women 5, Group 1–4).

God (Rabbi) sends diseases to a human being; we think no one can know what God brings to a human being. So, women who develop such diseases go to ‘Tsebel’-meaning holy water. (Women 11 Group 1–4).

## Discussion

The study aimed to explore enablers and barriers to BSE practice among women of in the North Shewa zone Oromia, Ethiopia. The findings of the study were discussed under five themes of enablers and three themes of barriers. The five themes of enablers were knowledge about BC, knowledge about BSE, experience of BSE practice, perceived susceptibility, and perceived benefit of BSE practice. The three themes of barriers were low knowledge of BSE practice, misconceptions about BSE practice, and fear of detecting BC.

The study results showed that understanding about BC and BSE were the main reason of why they do performed BSE. As confirmed from the discussions, most of the women who do practise BSE acknowledged good knowledge about BC, BSE and understanding of the technique. It is consistent with the studies conducted in Ethiopia, Modjo town, Iran, and Ruanda among women^35,40,41^. This gives the clue that when individuals have adequate knowledge about some disease problem and its consequence probability of to practice the preventive behaviour is high. Because of that knowledgeable respondents’ motivation to practice BSE.

The findings identified from the discussions as women who perceived they are susceptible to developing the disease have a higher degree of performing BSE and also, the majority of them believed that their risks are connected with having a family history of BC, breast enlargement in childhood period and supernatural power. Those women who believed BSE is important for early diagnosis and treatment are more tendency to perform it. Because their relatives and friends get the opportunity of early detection of BC (any breast problems) through self-examination. This finding was in agreement with the study conducted in Ethiopia, Hosanna town and UAE, in Ajman^28,29^ The possible justification, that women with a better understanding of the benefits of BSE, such as doing it regularly(monthly) are helping them to detect any changes (like lumps) before the health professionals are more willing to perform BSE.

The findings identified from the discussions as women who believe about BSE it’s important for early diagnosis and treatment for any breast abnormality were more tendency to practice it. This finding was in line with the studies conducted in different areas^29,42^. This study finding shows that the low knowledge of BSE practice was the main barriers for not practicing BSE. This finding implies that the lack of knowledge about BSE might prevent them from performing BSE, which might reduce chances of early detection of the disease. This finding was in line with the studies conducted in different areas^22,24,29^.

The other factor identified in this study was the misconception about BSE practice. This finding implies that the participants who had no history of a breast lump, symptoms they did not believe it important and afraid to talk about the practice of BSE. This finding was in line with the previous study conducted in Palestine^43^. The previous study revealed that many barriers for practicing BSE such as the perception of no disease threat, the lack of knowledge, the fear of detecting cancer, among others, that prevented participants from performing the BSE. These could have led to the low practice attitude among most of the participants who reported they had never practiced the BSE.

Finally, this study discovered that fear of detecting BC is another factor hindering the practice of BSE. This finding indicated that the majority of the women who do not practice BSE, were due to the fear of the outcome like chemotherapy, loss of hair and seriousness of BC. This finding was in line with the previous study conducted in Palestine^43^.

## Limitations of the study

Recall bias: Participants may have difficulty accurately recalling past experiences or events related to breast self-examination. This could result in incomplete or inaccurate information being provided during the discussion, affecting the validity of the study.

Limited scope: Qualitative studies often focus on exploring in-depth experiences and perspectives rather than providing quantitative measurements. While this approach provides rich insights, it may have limitations in terms of generalizability and statistical analysis**.**

## Conclusion

Generally, this study identified and described the enablers and barriers of BSE practice which are good knowledge of BC, good knowledge about BSE, experience of BSE, perceived susceptibility, perceived benefit of BSE practice, as enablers of BSE practice while, low knowledge of BSE, misconception about BSE and fear of detecting BC are the barriers of BSE practice. The study highlights the importance of addressing knowledge gaps, dispelling misconceptions, and addressing fears associated with BSE. Promoting knowledge about BC, providing accurate information about BSE, and enhancing women's perception of susceptibility and the benefits of early detection can contribute to increased BSE practice among women in the North Shewa Zone.

## Recommendation

Based on the findings of the study regarding the enablers and barriers to BSE practice, here are some recommendations for the concerned bodies.

North Shewa Zonal Health Office and the Regional Health Bureau

Develop and implement targeted health education programs: Design comprehensive health education programs that specifically focus on BC awareness, the importance of BSE, and proper techniques for conducting BSE.

Collaboration with local stakeholders: Establish partnerships with local organizations, community leaders, and influencers to enhance the reach and impact of health education initiatives. Engage these stakeholders in spreading awareness, organizing community events, and utilizing existing communication channels to disseminate information about BSE and BC.

Healthcare providers

Health care professionals should engage in the community to address the obstacles women face in practicing BSE.

Promote patient-centered care: Encourage healthcare providers to create a supportive and non-judgmental environment, where women feel comfortable discussing their concerns, misconceptions, and fears related to BSE and BC. Effective communication and empathy can help address barriers and encourage women to adopt BSE practices.

Cancer associations at the Regional Health Bureau

Support awareness campaigns: Collaborate with local health authorities and organizations to conduct awareness campaigns specifically targeting breast cancer and BSE. These campaigns can utilize various media channels, community events, and social platforms to disseminate information, raise awareness, and promote positive behavior change.

Support early detection services: Advocate for the availability of screening services, including clinical breast examinations and mammograms, in the North Shewa Zone. Collaborate with healthcare facilities to ensure access to these services, particularly for women who may require additional diagnostic procedures beyond BSE.

Researchers

They should be looks into a comprehensive community-based study at a national level on BC screening practices to plan an awareness creation program and a longitudinal study for a better outcome in all dimensions of breast screening practices.

## Data Availability

The original contributions presented in the study are included in the article/supplementary material, further inquiries can be directed to the corresponding author/s.

## Abbreviations

ACS: American Cancer Society
AOR: Adjusted odds ratio
BSE: Breast self-examination
BC: Breast cancer
CBE: Clinical breast examination
FGD: Focus group discussions
GLOBOCAN: Global Burden of Cancer
PI: Principal Investigator
SSA: Sub-Saharan Africa
WHO: World Health Organization
